# Explanatory Models of Genetics and Genetic Risk among a Selected Group of Students

**DOI:** 10.3389/fpubh.2016.00111

**Published:** 2016-06-06

**Authors:** Heather Honoré Goltz, Margo Bergman, Patricia Goodson

**Affiliations:** ^1^University of Houston-Downtown, Houston, TX, USA; ^2^Baylor College of Medicine, Houston, TX, USA; ^3^Milgard School of Business, University of Washington-Tacoma, Tacoma, WA, USA; ^4^Department of Health & Kinesiology, Texas A&M University, College Station, TX, USA

**Keywords:** health literacy, genetics, risk perception, genetic literacy, qualitative research, explanatory models

## Abstract

This exploratory qualitative study focuses on how college students conceptualize genetics and genetic risk, concepts essential for genetic literacy (GL) and genetic numeracy (GN), components of overall health literacy (HL). HL is dependent on both the background knowledge and culture of a patient, and lower HL is linked to increased morbidity and mortality for a number of chronic health conditions (e.g., diabetes and cancer). A purposive sample of 86 students from three Southwestern universities participated in eight focus groups. The sample ranged in age from 18 to 54 years, and comprised primarily of female (67.4%), single (74.4%), and non-White (57%) participants, none of whom were genetics/biology majors. A holistic-content approach revealed broad categories concerning participants’ explanatory models (EMs) of genetics and genetic risk. Participants’ EMs were grounded in highly contextualized narratives that only partially overlapped with biomedical models. While higher education levels should be associated with predominately knowledge-based EM of genetic risk, this study shows that even in well-educated populations cultural factors can dominate. Study findings reveal gaps in how this sample of young adults obtains, processes, and understands genetic/genomic concepts. Future studies should assess how individuals with low GL and GN obtain and process genetics and genetic risk information and incorporate this information into health decision making. Future work should also address the interaction of communication between health educators, providers, and genetic counselors, to increase patient understanding of genetic risk.

Accompanying the original 2001 publication of the Human Genome Project (HGP) in the journal *Nature*, Nobel laureate David Baltimore commented, “We are creating a world in which it will be imperative for each individual person to have sufficient scientific literacy to understand the new riches of knowledge, so that we can apply them wisely” (1). As genomics becomes more fully integrated into patient care (2), we must be assured that patients have the ability to apply the genetic information they receive and be confident that providers have the competencies necessary to bridge any potentially harmful gaps (3, 4). Nonetheless, over 10 years after Baltimore’s editorial, the question of what defines *sufficient* scientific literacy has not been fully answered, especially concerning genetics-related applications.

The National Center for Education Statistics defines *scientific literacy* as “the knowledge and understanding of scientific concepts and processes required for personal decision-making, participation in civic and cultural affairs, and economic productivity” (5). Conceptualizing genetic literacy (GL) starts with an understanding of scientific literacy and a related concept, *health literacy* (HL), which is “the degree to which individuals have the capacity to obtain, process, and understand basic health information and services needed to make appropriate health decisions” (6). The Institute of Medicine (IOM) estimates that nearly 50% of US adults have low HL. Thus, adults with low literacy may have issues with bringing up complex health problems, missing treatment doses, and navigating the entire medical system (7).

For genetics specifically, GL has been characterized as both “sufficient knowledge and appreciation of genetics principles to allow informed decision-making for personal well-being and effective participation in social decisions on genetic issues” (8) and the knowledge, skills, as well as the attitudes necessary for understanding of genetic information and for accessing genetic-based technologies and services (9, 10). The knowledge base for genetics necessarily requires that a patient possess both *literacy* (skills relating to reading and writing) and *numeracy* (skills relating to interpretation of numbers and numerical relationships), and these have been shown to be independent predictors of GL as well (10). To ensure that patients have this necessary level of understanding and decision-making skill requires an understanding of how they conceptualize genetics and genetic risk in order to identify and correct any misunderstandings and misconceptions that could affect the clinical process (11).

The current qualitative study was part of a larger research project exploring the relationship between genetic risk perceptions and sexual/reproductive decision-making in a college-age population. Based on participants’ responses to specific group interview questions, researchers were able to outline their explanatory models (EMs) of genetics and genetic risk. We then contrasted participants’ EM with extant biomedical models to highlight perceptions and knowledge, or participants’ GL. Based on our findings, we discuss implications for health disparities research, health education, and public health practice.

## Background

The most recent National Assessment of Adult Literacy (NAAL) results indicate over one-third (36%) of US adults have *below basic* or *basic* HL, and most minority subgroups had lower HL, on average, than their White counterparts (12). Lower HL is linked to increased morbidity and mortality for cardiovascular disease, diabetes, and cancer (13). Disparities in health status, care, and outcomes are well documented in racial/ethnic minorities having these conditions (14). As genomic and personalized medicine become more sophisticated, GL *proficiency* will become increasingly important for all patients, and these disparities will, therefore, become more marked among low HL groups.

It is a misconception that higher levels of general education, or even higher intelligence corresponds to higher general HL or health numeracy skills (15). Thirty percent of 2-year college graduates and 20% of 4-year college graduates have only basic quantitative literacy (16). Further research shows that college students lack general health numeracy skills (17) and, specifically, genetic numeracy (GN) or the numerical skills and conceptual knowledge required for interpretation and analysis of genomic and genetic risk (18). The lack of GN can affect a person’s ability to understand the risks of genetic disease, not only their own, but that of their offspring and future generations. Determining what factors shape a person’s genetic risk assessment can highlight where discrepancies between objective and subjective risk develop, discrepancies that have been shown to shape health behaviors (19).

In public health, describing people’s EM of illness facilitates exploring how they understand complex concepts (e.g., *health*, *genetic risk*) and/or communicate subjective experiences of illness (20–23). Eliciting these models is essential to uncover *cultural and conceptual knowledge* of specific health issues, an understudied HL dimension (12).

## Materials and Methods

### Participants

We recruited 86 students, at undergraduate and graduate level (≥18 years old), enrolled in 3 Southwestern US universities, to participate in focus group discussions. Two universities were located in major metropolitan areas, one, in a rural county. One university had a majority Caucasian enrollment, and the other two comprised mostly Hispanic, other minorities, and international student populations. Each site’s Institutional Review Board independently approved the study. We recruited participants *via* announcements during regularly scheduled non-science classes.

### Procedure

This study used focus group (FG) interviews for data collection; the method is particularly well suited to study social phenomena because *group interaction* often provides more data than individual interviews (24). Often the group dialog stimulates participants to discuss topics they had not previously contemplated due to habitual thinking or a lack of knowledge. Interaction in the group setting also allows conversations to develop based on shared values (25). Eight FGs took place between February 2007 and February 2008. Each university hosted at least two FGs; FGs comprised a minimum of 10 and maximum of 13 participants, lasted an average of 45 min, and were audiotaped with participants’ permission. The lead author served as recruiter and moderator.

### Instruments

The FG guide contained 15 open-ended questions and probes, organized into three sections (conceptualizing genetics, conceptualizing genetic risk, and operationalizing genetic risk in sexual/reproductive decision-making). Several questions were modified from the 2004 qualitative study by Lanie and colleagues (26). The guide underwent pilot testing and performed adequately (Appendix A). (The present paper will focus on findings from the first two sections, conceptualizing genetics and conceptualizing genetic risk.)

Participants also completed a one-sheet, anonymous demographic information form. Alongside demographic data, the form contained questions concerning exposure to genetic education, genetic or prenatal testing, and whether the participant had ever been involved in creating a family tree or written a family health history (FHH). Study participants were asked, also, to identify, from a list of various diseases, which ones they considered as “severe genetic disorders.”

Additionally, the form contained three questions modified from the 2004-*General Social Survey* assessing knowledge of genetic disorders, access to genetic testing information, and attitudes toward genetic screening. The *General Social Survey* is a biennial survey conducted by the National Opinion Research Center since 1972, this survey was conducted annually prior to 1994. This validated survey includes questions from a wide range of topics, assessing both demographics and attitudes of US residents, including items about overall awareness of genetic testing. The participants in our study were asked more detailed questions about where they accessed this information.

### Data Analysis

After transcribing the audio recordings of each FG, a single coder (Heather Honoré Goltz) analyzed the data using a *holistic-content* approach (27, 28). This process involved multiple readings of each transcript, identification of overarching themes in each interview, and subsequent comparison of themes across all interviews. Once initial coding was completed, a health education content expert (Patricia Goodson) reviewed the codes and resulting themes for coherence and fidelity of the qualitative data in the transcripts. A public health genetics content expert (Margo Bergman) then provided a final round of review of themes for coherence and comprehensiveness in relation to the interview guide questions.

For ease of understanding, we present findings by thematic content area. During analysis and interpretation, the authors attempted to preserve participants’ voices as much as possible and to establish data *correspondence* and *coherence* by presenting direct quotes from study participants and mirroring their language in interpreting findings (29).

## Findings

### Demographics

The sample (*N* = 86; FGs = 8) was diverse in age, race/ethnicity, annual household income, and religious preference (Table 1).

**Table 1:** **Participant demographics**.

Demographics	Site 1	Site 2	Site 3	All sites
**Gender**				
Male	22	1	5	28 (32.6%)
Female	14	19	25	58 (67.4%)
**Race/ethnicity**				
Asian/Pacific Islander	2	2	4	8 (9.3%)
Black/African American	3	6	3	12 (14.0%)
Hispanic/Latino	18	2	5	25 (29.1%)
White/Caucasian	11	9	17	37 (43.0%)
Other	2	1	1	4 (4.7%)
Average age (SD; range)	28.8 years (8.8 years; 19–54 years)	31.1 years (8.6 years; 19–50 years)	24.7 years (5.7 years; 19–44 years)	28 years (8.1 years; 19–54 years)
**Marital status**				
Single, never married	24	13	27	64 (74.4%)
Divorced	1	1	1	3 (3.5%)
Married	11	5	2	18 (20.9%)
Widowed	0	1	0	1 (1.2%)
**Have biological children?**				
Yes	9	4	3	16 (18.6%)
No	27	16	27	70 (81.4%)
**Religious preference**				
Catholicism	16	3	10	29 (33.7%)
Christian, other	13	9	11	33 (38.4%)
Hinduism	0	2	0	2 (2.3%)
Judaism	0	0	1	1 (1.2%)
Protestant	2	2	3	7 (8.1%)
Other	4	4	4	12 (14.0%)
**Current educational status**				
Undergraduate	25	0	13	38 (44.2%)
Graduate	11	20	17	48 (55.8%)
**Annual income (student’s or parent’s)**				
$0–$14,999	7	4	7	18 (21.2%)
$15,000–$29,999	8	3	7	18 (21.2%)
$30,000–$44,999	5	3	5	13 (15.3%)
$45,000–$59,999	2	2	1	5 (5.9%)
$60,000–$74,999	4	0	0	4 (4.7%)
$75,000+	10	7	10	27 (31.8%)
**Highest degree earned**				
GED/HS diploma	17	0	11	28 (32.9%)
Associate’s degree	7	0	1	8 (9.4%)
Bachelor’s degree	2	17	4	23 (27.1%)
Master’s degree	10	3	13	26 (30.6%)

**Table 2:** **Responses to knowledge, attitude, and experience questions**.

Knowledge, attitudes, and experiences				
	**Site 1**	**Site 2**	**Site 3**	**All sites**
Ever had genetics course?				
Yes	2	5	8	15 (17.6%)
No	34	14	22	70 (82.4%)
Ever had course with genetics information?				
Yes	16	15	20	51 (59.3%)
No	20	4	10	34 (39.5%)
I don’t know	0	1	0	1 (1.2%)
Ever charted family tree (genealogy)?				
Yes	14	12	11	37 (43.0%)
No	22	7	19	48 (55.8%)
I don’t know	0	1	0	1 (1.2%)
Ever charted family health history?				
Yes	10	6	11	27 (31.8%)
No	26	13	19	58 (68.2%)
Ever had prenatal testing?				
Yes	2	1	1	4 (5.9%)
No	30	13	17	60 (88.2%)
I don’t know	1	0	1	2 (2.9%)
I do not want to answer	1	1	0	2 (2.9%)
Ever had genetic testing?				
Yes	1	1	3	5 (5.8%)
No	33	19	26	78 (90.7%)
I don’t know	2	0	1	3 (3.5%)
If you/your partner pregnant, would you want test for serious genetic disorders?				
Yes	22	15	19	56 (68.3%)
No	11	3	4	18 (22.0%)
I don’t know	3	1	4	8 (9.8%)
How much have you heard or read about genetic testing?				
A great deal	0	3	2	5 (6.0%)
Not very much	31	17	23	71 (84.5%)
Nothing at all	4	0	4	8 (9.5%)
Do you think genetic screening will do …?				
More good than harm	22	10	9	41 (48.8%)
More harm than good	2	0	0	2 (2.4%)
It depends	9	9	20	38 (45.2%)
I don’t know	2	0	1	3 (3.6%)

Participants ranged in age from 19 to 54 (M = 28.0 years, SD = 8.1 years); most of them were females (67.4%). The sample contained “White/Caucasian” (43%), “Hispanic/Latino” (29.1%), and “Black/African American” (14%) participants. Regarding religious preference, most declared themselves “Christians” (88.1%). The majority were undergraduate students (81.4%).

Participants varied according to marital and parenting status, and regarding exposure to genetic information and services. Although most participants were “Single, Never Married” (74.4%), and did not have biological children (81.4%), almost one in five (18.6%), including some “Single, Never Married,” had biological children. Less than 6% reported ever having undergone genetic testing, while 16.7% of females with children underwent prenatal testing during pregnancy. Further, no participants were biology or genetics majors; most had never taken a genetics course (82.4%) nor charted their FHH (68.2%).

### Explanatory Models

The findings below are organized in two sections. The first section discusses themes related to participants’ EM relating to genetics. The second section discusses themes related to their EM models of genetic risk. Each EM model consists of different factors, including the intersections of psychosocial, cultural, environmental, and spiritual factors. Psychosocial factors include attitudes, beliefs, finances, family dynamics, and social supports. Cultural factors include age, generation, and exposure to technology, race, or ethnicity. Environmental factors include community, resources, policies, and regional variation. Spiritual factors include religiosity, denomination, and orthodoxy.

### EM Models of Genetics

#### Genetic Literacy

When asked to describe “what comes to mind” when “genetics” is mentioned or what makes something “genetic,” most participants’ responses focused on listing genetic concepts, characteristics, technologies, or human biological relationships (Appendix B). Several terms that participants listed – “DNA,” “mutations,” and “dominant traits” – are commonly used in genetic discourse. Yet, few respondents defined these terms or described biological mechanisms and gene–environment interactions.

Only one participant provided a more sophisticated explanation of the biomedical EM. This participant described mechanisms underlying genetic mutations as “alteration of a gene” and “chromosomes (that) didn’t match up” (Asian male, site 2). This same participant provided an explanation for hemophilia inheritance:

It’s a dominant or recessive character of ah, the, the base pair, the gene base pair, you have. For example, hemophilia would show up in the female if both the X have got the hemophilic factor, otherwise, in a male, even if one X has H, the male would show up to be hemophiliac.

No other participants provided this depth of explanation and the quote above was the only FG participant with previous biomedical training. Thus, use of specific scientific terminology within the EMs of genetics of other participants may be more indicative of chronic exposure to these concepts in popular media rather than exposure to biomedical conceptual models. This finding is supported by responses from the demographic data sheets, where Internet resources were the most commonly cited source of genetic information.

Also supporting this notion that participants’ knowledge of genetic concepts is superficial were data from the demographic sheets related to recognizing “genetic disorders.” Participants were asked to identify “severe genetic disorders” from a list of different diseases, including genetic disorders, non-genetic disorders, disorders having ambiguous genetic links, and health conditions involving gene–environment interaction (e.g., genomic disorders). Correctly identified disorders included Down’s syndrome (81.4%), cystic fibrosis (58.1%), and sickle cell anemia/disease (55.8%), conditions that receive routine media coverage (30–34).

A rather disturbing finding was that almost one in five participants (19.8%) also labeled HIV/AIDS a “severe genetic disorder.” One explanation for this finding is that the molecular biology of HIV/AIDS and maternal–child transmission continue to be discussed in popular media. If participants are transposing limited understanding of viral genetics onto understanding of HIV during pregnancy, they may be combining these mechanisms within their EM. In essence, an EM of HIV transmission with genetic attribution emerged from this sample. EM attributing hereditary, not infectious, causes to AIDS may be problematic vis-à-vis the prevailing paradigm of HIV as an infectious agent.

#### Family Health History

Major themes with respect to FHH included *DNA as destiny* and *family as genetic mechanism* (i.e., *n* = 27 or 52% of codes). Participants shared many personal stories related to FHH. Their narratives firmly placed families, not genes, as the unit and mechanism of inheritance. Familial, and thus genetic, traits could be physical or emotional/mental. These traits were “passed down” or “inherited from someone above you in the family line” (Hispanic female, site 2).

Based on participants’ responses, genetic characteristics could be “good” or “not always negative.” However, they were “predetermined” and resulting from familial “predispositions.” A number of participants believed the presence of a particular trait in the “bloodline” would virtually *guarantee* the emergence of a disease state; few respondents expressed belief in asymptomatic carrier states. Thus, FHH emerged as a strong and invariant determinant of health outcomes for individuals, within participants’ EM.

#### Psychosocial and Environmental Factors

Participants also included non-genetic factors in their EM of genetics. Several referred to prevention-focused health care, dietary modification, and lifestyle or environmental factors (e.g., economics, access to health services) as determining whether genetic traits manifest themselves. In response to the question “How do genetic traits manifest?” one participant stated, “well, if you’re poor you’re not gonna have access to healthy food, the Internet or preventative (care), you know, (or) go to the doctor if you’re starting to get sick or any of those things” (White female, site 1), showing a linkage in her mind between these items and genetic disease. In another case, a participant identified spirituality in response to this question. When asked “How do genetic traits manifest?” she expects spiritual beliefs would aid one’s coping ability, thus decreasing the likelihood that a genetic-linked disorder (in this case depression) might manifest or reducing its impact on daily living:

I would say your spirituality because if you look at something like depression and how it manifests in, your spiritual life might help you cope, or … manage … everyday life. (Black female, site 3)

Our sample’s genetics EM, therefore, included many of the psychosocial, cultural, environmental, and spiritual factors mentioned above as influences on genetic expression, rather than purely biological mechanisms. Such a model suggests that participants may be more prepared to incorporate concepts related to public health genomics, epigenetics, and genomic medicine, than previously assumed.

#### Attitudes toward Genetic Disorders and Testing

Most participants reported – on the demographic data sheet – they never underwent genetic or prenatal testing. This finding aligned itself with their having read or heard “not very much” (84.5%), or “nothing at all” (9.5%) about genetic testing. In contrast, when asked if “genetic screening (in general, not for them, specifically) will do more good than harm, or more harm than good,” participants’ attitudes toward genetic screening were either neutral (45.2%) or positive (48.8%).

Participants were asked, “Are some genetic disorders better to have than others?” Several rated genetic disorders perceived as “manageable,” “preventable,” exhibiting lower “severity,” or having lower impact on functioning as “better.” Specific examples included chronic diseases (e.g., diabetes), cancers, and sickle cell disease (i.e., due to anti-malaria properties). Additionally, participants frequently juxtaposed mental and physical disorders that were genetic in origin and implied a ranking in which physical disorders were more acceptable than mental ones. For example, genetic and genetic-linked disorders, such as “Down’s syndrome” and “schizophrenia,” which may impact cognitive and affective functioning, were often perceived as being more severe and debilitating and thus, rated “worse” than disorders, such as “Spina Bifida” or “Sickle Cell.” Participants also rated genetic-linked behavioral health conditions, such as “alcoholism” and “drug abuse,” as potentially more severe and debilitating than “dwarfism” or “Multiple Sclerosis.”

### EM Models of Genetic Risk

#### Genetic Risk and Numeracy

Participants defined “genetic risk” in a variety of ways. Some definitions were vague: “taking a big chance” or “the risk of inheriting something” (Appendix C). Others relied on case studies to communicate more complex notions. Approximately one-half of informants thought about genetic risk in relation to decisions to have children. Other participants conceptualized genetic risk in terms of dating or marrying, in terms of FHH, a disorder’s perceived severity, or personal experience with a specific disorder.

Several participants reported as not being concerned about their potential genetic risk due to being *younger* or being guided by *faith*. Those who were younger believed they were *risk takers*. One female participant admitted that she had been more of a risk taker as a young adult because she had less knowledge of genetic health issues in her family; however, she admitted her perspective changed considerably as she aged.

When I was younger I heard people talking about hypertension and stroke and this and that and I really didn’t know what it was until I started to go to the doctor … do you have any history of this and this and this and I really didn’t know, so I asked my mom and she was like yes, yes, yes. So, when I did that and then I realized, you know, I’m at risk for a lot of things, but I guess genetics are. … So when you’re young you really don’t see the difference, but you’ve got to … but now that I’m older it’s really kinda scary, you know, am I gonna get diabetes or these things … kind of weird. (Hispanic female, site 1)

Participants who relied on religion or faith for guidance were also “more willing” to take risks in childbearing. Another female participant (White female, site 1) stated, when asked whether she would undergo genetic testing, “What do I do with that information? Is it going to stop me from wanting to have a child … it didn’t and all of that, we kind of defaulted to the faith thing.”

#### Genetic Numeracy

Medical and health professionals often operationalize genetic risk using numbers, particularly frequencies, percentages, and probabilities. When asked how they interpreted genetic risk in probability terms (e.g., a one-in-four chance), responses indicated potential lack of clarity in the construct, despite its apparent numeric precision:

One in four out of like, the world? One in four out of the US? … so the one out of four … you still have to nail down … what is that based upon? (White male, site 1)

Further, participants interpreted biomedical explanations of genetic risk in terms of absolute risk rather than relative, fluid, and complex calculations. One participant provided this example:

I know a lady who they were telling her that she, her child … wouldn’t have this disorder … it was a brand new mutation it wasn’t inherited, and so (her risk) was like one out of 70,000. So her first child had it, and she had a second child because she thought, you know, one out of 70,000, she’s not gonna get it … but she had another one with the disorder too. (White female, site 2)

Here, the participant is discussing how her friend is perceiving the risk of a genetic disorder as absolute risk over a lifetime, rather than a relative risk, whereas in reality, the risk calculation for a subsequent child changes after a previous child is born with the disease. Consequently, participants’ understanding of probability-type risk information appears fixed when it is received, rather than being updated with new information. When participants must make additional health decisions, in this case childbearing, they rely on previous outcomes (i.e., the past outcome was “good” or “bad”) to frame their *current* risk and make decisions accordingly. This also highlights a disconnect in understanding of the participants between overall risk and individual risk. This finding highlights potential communication issues when providers translate concepts of genetic inheritance into clients’ colloquial language.

#### Proximity

Several FG participants expressed that finding out someone in their family had a genetic disorder would increase their awareness concerning the disorder, and motivate them to perform genetic testing, regular check-ups, or lifestyle changes. Some participants qualified this change in awareness of personal risk in terms of *proximity* of the disorder in the family tree, and of the disorder’s *severity*. Proximity was defined as “how close(ly)” the participant related (biologically) to the affected relative. *Immediate* relatives, parents, grandparents, siblings, aunts, uncles, and first cousins, were considered *close*. Yet *closeness* was also defined in terms of frequency of interaction. One participant stated:

It depends on how close in your family it is … my great aunt is … Schizophrenic … but because she is my great aunt I don’t feel really nervous about it … I’m hoping I’m not schizophrenic, which is possible, but um, I guess I’m not too worried, but if I had known her more when I was younger … (Black male, site 1)

Similarly, another participant stated:

A great cousin, you know, that I’ve never met, you know, that’s not gonna affect me as much as a parent versus a grandparent or a sibling. (Black female, site 2)

Similar to the EM for genetics, the EM of genetic risk extended beyond purely biological mechanisms and probabilities. Participants conceptualized genetic risk across biological, affective, attitudinal, and perceptual domains, as well. Their EM of genetic risk contains highly contextualized personal, familial, or social narratives rather than the mathematical models presented in scientific literature. This finding has important implications for health-care providers as they attempt to explain genetic risk to patients/clients: in many instances it might be more useful to frame genetic risk in terms of the clients’ affect (emotions), attitudes, and perceptions, before focusing on numerical probabilities, chances, and percentages.

#### Perceived Severity

“Severity” refers to where – along a continuum – an individual’s signs, symptoms, or level of impairment due to a genetic condition may fall. Perceptions of severity ranged from not affected to completely disabled. Participants relied on their perceptions to compare genetic disorders and assess severity:

Severity of it … some of those (genetic disorders) are really disabling … Like, if you were born with Down’s syndrome, you can’t do anything to kind of control it, as much as if you develop diabetes or something like that which you can control by food and insulin. (White female, site 3)

Thus, perceptions of a genetic condition’s severity (defined more as perceived impact than medically objective indicators), appear to play an important role within the sample’s EM of genetic risk. Perceived severity relates to acceptability as discussed above in participants’ EMs of genetics, displaying an implied ranking of physical over mental genetic disease. If a disease has a known intervention, then the perceived severity is lower than those conditions without obvious treatments. However, mental disorders, such as bipolar disorder or schizophrenia, are perceived as most severe, regardless of treatment availability.

## Discussion

We were interested in exploring perceptions of genetics and genetic risk in what we thought was a best-case scenario – a well-educated population of reproductive age. Almost 60% of our sample had obtained at least a bachelor’s degree at the time of this study, with a further 9% having completed at least 2 years of post-secondary education. We expected this group would exhibit understandings of genetics that mimicked the biomedical models, given their college education. However, we found instead that this population based their EMs of genetics and genetic risk on family history and relationships, media, cultural norms, and religious influences.

Overall, participants exhibited some oral and knowledge-based HL when interpreting and discussing genetic risk information, even when they were unfamiliar with their mechanisms. Additionally, this sample indicated a belief in the concepts represented by epigenetics rather than genetic determinism, even if they did not use this terminology. Similar to Lanie and colleagues (26), our study participants’ risk perceptions and understanding of risk information appear to be grounded more in highly contextualized personal, familial, or social narratives than in scientific literacy [over one quarter (26.7%) of participants reported that they, a close friend, or a relative were affected by genetic disorders]. All participants’ EM overlapped somewhat with biomedical models; however, their models primarily contained affective, moral, socioeconomic, and environmental components that participants relied on for hypothetical or real genetic health decision-making.

These findings reveal that, in spite of their level of educational attainment, the students’ EMs are similar to those in lay populations. Specifically, student and laypersons’ EM is more commonly influenced by personal or familial experiences with genetic or genomic diseases and cues to action from popular media than by biomedical models (35). Additional research suggests that GN, and consequently, GL, do not appear to be affected by non-targeted higher education (36). Thus, an internal EM of genetics and genetic risk will likely reflect biomedical models only after prolonged exposure to specific, specialized training in such models.

### Implications

In our sample, participants misinterpreted numerical genetic risk, visualizing each potential genetic risk as an independent event, rather than a dependent risk calculation. As a result, GN may not influence risk-bearing decisions to the same extent as perceptions of the disorder’s severity or seriousness and its perceived impact on potential children. These perceptions are sometimes linked to what people have seen in others, or experienced themselves (37). If, for instance, an individual socializes with someone during pro-dromal periods of a genetic or genetic-linked disorder (e.g., Huntington’s), his or her initial impressions may be that the disorder is not as severe as the “more visible” genetic disorders, such as Down syndrome. This supports research that shows this type of “framing” effect increases biased health decision-making (38) and reduces understanding of genetic risk (39).

Differences in biomedical versus personal EM of genetics and genetic risk require health-care providers and the public health workforce to invest in understanding these personal models for the successful and beneficial implementation of public health genomics and genetic services. One possible method for such successful implementation is generating increasingly accurate explanations of genetic materials in the public media, which already tend to take into account the non-numerical and non-biological dimensions of GL elicited in this study. This undertaking becomes particularly imperative, as genetic/genomic testing is increasingly incorporated into routine medical care and accessible *via* direct-to-consumer tests, thereby providing complex genetic information to population groups with relatively low GL. Further, the American Public Health Association has developed a policy statement containing comprehensive recommendations for strengthening genetic and genomic literacy among the general public and public health professionals to aid in these efforts (40).

In our study, participant’s personal connections and FHHs were strong motivators of their understandings of genetics. Knowledge of FHHs alone, however, is not sufficient for increasing GN (41). FHHs in conjunction with genetic counseling – a common intersection point between biomedical and personal models of genetics and genetic risk especially in the case of genetic disease – could increase understanding. However, the supply of genetic counselors is already insufficient for the current workload, much less for improving the GL of the entire population (42, 43). Therefore, in order to take advantage of the potential of FHH to reduce misinformation stemming from personal EMs of genetics/genetic risk and infuse the correct biomedical conceptualizations into these EM models, additional sources of health education, such as Internet tools, discussions with primary care providers, and health educators (4, 11, 44, 45) are vital. Health educators are more numerous than genetic counselors and have a more focused mission than primary care doctors; they are, therefore, ideal professionals to undertake this important role. Further, research should also be performed to determine how various visual and graphical presentations of probability could compensate for low GN in these educational contexts.

### Limitations

From a positivist perspective, this study contained some limitations related to sampling (e.g., technique, size, and generalizability). However, these issues, particularly generalizability and statistical representation, are not the purview of qualitative inquiry. Rather, maximum variability in themes extracted is the goal of qualitative methodologies (46). Our sample comprised mostly female college students; thus, their knowledge, attitudes, and potential behaviors may not reflect the full range of individual beliefs, or extrapolate to those found in the general public. Further, personal experience, especially with mental (e.g., Schizophrenia) and cognitive disorders (e.g., Down syndrome), may have exerted a greater influence on participants’ attitudes than noted and may even have affected decisions to take part in the study. Even so, the limited qualitative research published in this area subsequent to our study provides support for our findings related to personal EM of genetics or genetic risk (47–49).

### Conclusion

Our findings reveal gaps in how a sample of young adults obtains, processes, and understands genetics/genomics and risk. These gaps can result in misunderstandings of the sources of genetic risk, the implications of these risks, subsequent decisions, and resulting health behaviors (9, 11, 13, 14, 47–49). While participants in this study are among the most literate group in the US population, HL, GL, and GN represent a specialized range of knowledge, including *print literacy* (*reading*, *writing*, and *numeracy*), *oral literacy* (*listening* and *speaking*), and cultural/conceptual knowledge within a health context (7, 11). Such skills may be elusive even for those with a college education, as 3% of NAAL participants with Bachelor’s degrees and 15% with high school diplomas had below basic HL (44). This poses serious challenges for education systems to improve these skills and increase HL, GL, and GN. Higher education institutions have a responsibility to lead the way in improving HL by developing evidence-based programs across the education spectrum (7). One potential solution is the concept of plain language, or “communications that engage and are accessible to the intended audience” (50). A recent randomized trial of “plain language” versus “standard” language for self-injecting drug instructions shows that participants who receive plain language better understand and demonstrate the proper usage of the injecting pen (51).

Public health educators and genetic health professionals may need different models for educating clients of different ages and cultures about genetic issues or for assessing knowledge and understanding. Lay-level GL may require moving beyond oral and print literacy toward assessing and adopting lay people’s EM of genetic disorders and risk. Several studies have explored EM models of heritable disorders (e.g., breast cancer) in diverse populations to assess cultural/conceptual knowledge and medical decision-making (11, 52, 53). Understanding these diverse EMs is a necessary step toward achieving concordance between lay and biomedical EM, in an effort to improve prevention and care for people with genetic disorders.

The original goal of the HGP was to “greatly increase our understanding of human biology and allow rapid progress to occur in the diagnosis and ultimate control of many human diseases” (54). We have made vast progress on the first part of this goal, the biology, but control of disease requires determining and increasing the necessary HL skills of patients as well (55). GL and GN represent an area ripe for reconceptualization and innovative public health education initiatives. Promoting knowledge of etiology and genetic mechanisms may not be as important as facilitating understanding of risk factors and the implications of risk for inheriting specific disorders. Individual and social attitudes concerning specific genetic or genomic diseases and their implications for living and quality of life may be promising targets for educational interventions. Future studies should assess how individuals with low GL and from various racial/ethnic minority groups obtain and process genetic information, access and navigate services, and incorporate risk perceptions into health-related decision-making.

## Author Contributions

HG designed the study, served as the FG facilitator, and analyzed the data. She also was the lead author in drafting the initial manuscript. MB contributed to data analysis and preparation of the manuscript’s Introduction, Discussion, and Conclusion sections. PG contributed to study design, data collection, and data analysis. She also provided feedback on manuscript revisions.

## Conflict of Interest Statement

The authors declare that the research was conducted in the absence of any commercial or financial relationships that could be construed as a potential conflict of interest.

## Appendix A

### Interview Guide for Focus Groups

#### Defining genetics:

1. When I say “genetics,” what, if anything, comes to mind? [modified from Lanie et al. (26)]
2. When someone says that an ability, behavior, characteristic, or problem is “genetic,” what does this mean? [modified from Lanie et al. (26)]
3. Are some genetic disorders “better” to have than others?
4. When you need or want genetic information, where do you get it?
5. What factors may influence whether someone will manifest a genetic ability, behavior, or characteristic?

#### Defining “genetic risk”:

6 When I say the term “genetic risk,” what, if anything, comes to mind?
7 If you knew that someone in your family had a genetic disorder or a health problem related to genetics, how would you feel?
  Probe = without getting into specific issues, would you feel like you are also at risk of developing the same genetic disorder or health problem?
8 Which of the following phrases makes more sense to you and why?
  “Someone has a 25% chance of developing a genetic disorder.”
  “Someone has a 1 in 4 chance of developing a genetic disorder.”
  “Someone has a 75% chance of not developing a genetic disorder.”
  “Someone has a 3 in 4 chance of not developing a genetic disorder.”
  Probe = which of these statements sounds negative?
  Probe = which of these statements sounds positive?

## Appendix B

### Sample Quotations from PGRID Focus Group Interviews: Conceptualizing Genetics

#### Genetic literacy

DNA, genes, chromosomes, hair/eye color, XX/XY, dominant/recessive traits, science, stem cells, cloning, the genome project, heredity, Mendel’s peas, cells, biology, heredity

#### Family health history (FHH)

“further back (in the line) wouldn’t affect me,” “more immediate (family) would affect me,” “inherited from someone above you,” “it runs in the family,” “You got it from your mom,” “(you get it from) someone who you know, your parents” “Your past family’s history, like your medical history, like what diseases your family has”

#### Psychosocial and environmental factors

“Well like, diabetes, that’s something that if you know it’s in your family, you can monitor it and watch your sugar levels and stuff and I’m sure in some instances, it’s inevitable that you might get it, but in some instances, it may be avoidable if you watch your, you know, sugar and all that” “high blood pressure, cholesterol, those are some things that science is telling us now that there’s some genetic link and plus you can do something about it” “And even lung cancer, I mean if you know that’s, that’s in your family, then you know, you can make the decision to start smoking or not”

#### Attitudes toward genetic disorders and testing

“some (genetic disorders) are less life debilitating,” “predetermined,” “hard to fix,” “can’t control,” predisposed,” “It’s a problem that, that you have no control over” “Yes there are some that are better. Maybe because there’s some that you can do something about it. Like, maybe like your weight, you can do something, but then other things, like sickle cell might be a little more difficult for you to change” “There’s better medical technology out there for different diseases and genetic things that can help you later in life. It depends what research is going on in those areas to better your lifestyle”

## Appendix C

### Sample Quotations from PGRID Focus Group Interviews: Conceptualizing Genetic Risk

#### Defining “Genetic Risk”

“likelihood of getting” or “having a child with a trait;” “high risk,” “elevated risk,” “the chance of (something) happening;” “marrying someone with a risk” “I think that (there is an) elevated risk (for) women getting pregnant after, what is it in their forties, 45 (years old)?” “I guess it all depends … I’m almost 35 and I’m trying to get pregnant and I think if you’re willing to take that risk and I think I am and I think that regardless, I guess, if I’m 25 or I’m 35 or I’m 45, whatever God’s gonna send me, God’s gonna send me, regardless of what I do, so I don’t really think of that as risk” “I was thinking I have a cousin who is a carrier for hemophilia and just the risk that she took in the possibility of having a boy but she ended up with two girls, but yeah just taking that risk”

#### Genetic numeracy

“I was thinking about my brother and his wife … Because um, of her earlier pregnancy, she (found out she) was a carrier of this chromosome dysfunction factor, and if she were to pass it on, the high propensity for them to, you know, 50/50 chance (of having a child with the disorder), but they’ve had 2 subsequent children and they’ve had no problem” “I know one of my friends, when she was, when her mother was pregnant with her, she had a 50% chance of being Down’s syndrome but her parents decided to go ahead and still have her but she didn’t have it, but that’s a risk because you might have a child who has Down’s syndrome”

#### Genetic risk perceptions

“I have two first cousins who are … they are the weird … I grew up with them I mean, I played with them everyday and then in time it (schizophrenia) became full blown it was like a different person. It makes you kind of nervous just thinking for me because they’re so close to me in relation, they’re my cousins, of if I had a kid there’s a possibility …”

#### Perceived severity

“I think you also have to weigh into that the severity of the genetic disorder because, you know, we talked about sickle cell or you know, dwarfism or something to that nature. You know, is that more severe as say Multiple Sclerosis or um, some type of like Down’s syndrome?, but that’s still a part of the issue, you’re still saying I’m gonna see what happens, but you might take a little more time, and consideration because of the genetic disorder” “In my, in my family specifically all of the girls in my family, except for me, suffer from either bipolar or depression, and all the guys in the family have ADD … but talking to my sister … there have been countless times we’ve had conversations about ‘well I have to tell him (her boyfriend) that I’m bipolar, like, obviously that’s something that he’s gonna need to know.’ And it’s never just a ‘oh yeah I’m bipolar, so what do you want to have for dinner?”
